# New Appliances and Things Medical

**Published:** 1895-12-28

**Authors:** 


### NEW APPLIANCES AND THINGS MEDICAL.

[We shall be glad to receive, at our Office, 428, Strand, London, W.C., from the manufacturers, specimens of all new preparations and appliances, which may be brought out from time to time.]

### THE EXCELSIOR BED SUPPORT.

We have received from the "Bed Support Company," The Baths, Coventry, a specimen of the "Excelsior" bed support, a new and very practically useful invention for preventing the patient slipping down in the bed, a difficulty particularly present when a back rest is in use. The idea originated with one of the ward sisters in the Birmingham General Hospital, in which institution it is much in demand. The support consists of a piece of wire mattress webbing fixed to a light wooden frame with brackets, the length of the frame being two feet nine inches, and the height of the whole thing five and a-half inches. This long, narrow rest is placed so that the thighs of the sick person may rest upon it, and is secured to the head of the bedstead by two straps of firm webbing, which keep it in position. If a back rest is used the patient will thus be comfortably and securely supported, and all possibility of sliding gradually down obviated. The wire support is protected by a waterproof-covered pad. The price complete is 15s., and this is the only make at present ready for sale. More elaborately finished articles will be supplied by and by; but the one which we have seen is very well made and complete, and likely to satisfy every requirement for hospital and ordinary use. The practical experience which has resulted in the invention of this support is a testimonial in itself, and we have no doubt that wherever taken into use (and it is being now supplied to several hospitals, and where it is giving general satisfaction) both patients and nurses will find it a welcome boon, especially for cases of heart or lung disease, where an upright position has to be maintained.

